# Mechanical and Thermal Characteristics of Films from Glycerol Mixed Emulsified Carnauba Wax/Polyvinyl Alcohol

**DOI:** 10.3390/polym16213024

**Published:** 2024-10-28

**Authors:** Abodunrin Tirmidhi Tijani, Tawakalt Ayodele, Musiliu Liadi, Niloy Chandra Sarker, Ademola Hammed

**Affiliations:** 1 Environmental and Conservation Sciences, North Dakota State University, Fargo, ND 58102, USA; abodunrin.tijani@ndsu.edu (A.T.T.); tawakalt.ayodele@ndsu.edu (T.A.); liadi.musiliu@ndsu.edu (M.L.); 2 Agricultural and Biosystems Engineering, North Dakota State University, Fargo, ND 58102, USA; niloy.sarker@ndsu.edu

**Keywords:** plasticizers, biodegradable film, tensile properties, differential scanning calorimetry, emulsified wax

## Abstract

Poly(vinyl alcohol) (PVA)-based films have drawn significant attention owing to their potential applications in various industries. The application of wax to PVA films enhanced their resistance to dissolution and water infiltration. Nevertheless, waxed PVA films often exhibit inadequate mechanical properties owing to crack formation. In this study, we evaluated the impact of glycerol as a plasticizer in varying concentrations of Carnauba wax (CW). The addition of glycerol to the PVA/CW blend led to enhanced mechanical properties compared to the blend without glycerol. The functional group and morphology of the blends confirm glycerol compatibility with PVA/CW films. Glycerol was fully dispersed to form a consistent polymer matrix and equally improved the film’s contact angle. Furthermore, the thermal property from differential scanning calorimetry and thermogravimetric analysis highlights the plasticizing effect of glycerol in PVA/CW films, potentially broadening their use in food packaging and wrapping applications.

## 1. Introduction

Biopolymers in recent years have attracted significant interest. Their use has spread across industries, from agriculture and packaging to textiles and medical applications. Biopolymers are non-toxic, biodegradable and biocompatible in nature, thereby offering extensive applications. Similarly, the global biopolymer market is witnessing huge growth, with its market value expected to surpass USD 25 billion by 2027 [1]. This increasing demand for biopolymers is attributed to the high consumer need for sustainable, eco-friendly materials. Furthermore, biodegradable material sources are non-fossils, thereby reducing the reliance on fossil fuels. Many of these biopolymers can naturally decompose into harmless substances, unlike petroleum-based plastics. Despite the promising potential of biopolymers, there are several challenges limiting their usage. Poor mechanical properties (e.g., strength and durability) are one of the major challenges restricting the use of biopolymers in demanding applications. Additionally, variability in performance often occurs in biopolymers due to the source of the material used. This raises concerns about its consistency and scalability in large-scale production.

Poly(vinyl alcohol) (PVA) is among the popular synthetic polymers for biomaterial development. PVA is water-soluble and non-toxic, making it suitable for biopolymer production [2]. When combined with other materials, PVA composites are found useful for drug delivery applications [3,4]. Furthermore, the addition of natural emulsified waxes, such as carnauba wax (CW) and beeswax (BW), has greatly expanded the range of applications for biopolymer-based products in packaging and transdermal drug delivery systems [5,6,7]. The incorporation of emulsified waxes into films influences the mechanical properties of films (e.g., tensile strength and flexibility) and causes cracking because of reduced intermolecular interactions [8]. These films’ properties could be improved via plasticizing [9]. Plasticizers often used for biopolymer films are hydrophilic polyols (e.g., glycerol, xylitol, polyethylene glycol, and sorbitol) [10]. Films containing plasticizers become more flexible and hydrophilic [11,12]. High molecular weight plasticizer increases thermal resistance, tensile strength, and storage. PVA, in itself, could not stand water, making it unfit for packaging scenarios that could involve water contact. Mixing PVA with other materials to form a composite could result in better water resilience. However, other properties like the strength and stability of films relative to temperature could be negatively impacted. Hence, our ongoing effort to improve and adapt PVA-based film for packaging involves the addition of other materials, necessitating conducting this research to understand the changes in resulting film properties.

This present study aimed to prepare environmentally friendly PVA composite films with improved mechanical and thermal properties. More specifically, the purpose of this research was to develop biodegradable films composed of PVA and CW using the casting solution technique. The effect of emulsified CW/SA and glycerol as a plasticizer at different concentrations on the hydrophobicity, tensile, and thermal properties of PVA films was investigated.

## 2. Materials and Methods

### 2.1. Materials

PVA (Average MW: 205,000 g/mol) and Glycerol (G) were obtained from Sigma-Aldrich Chemicals, St. Louis, MO, USA, carnauba wax (CW) flakes were from Thermo Fisher Scientific, Waltham, MA, USA, polysorbate-20 and stearic acid (SA) obtained from VWR Chemicals, Solon, OH, USA were used for the emulsion formulation.

### 2.2. Film Preparation

The composite films followed the formulation in Table 1, as reported in the previous study, with slight modifications [13]. A total of 2.5% (*w*/*v*) PVA pellets were dissolved in 45 mL of hot distilled water with continuous stirring at a speed of 600–800 rpm for 1 h at 100 °C in a hot water bath. PVA was completely dissolved; thereafter, varying concentrations of glycerol were incorporated into the solution and allowed to mix thoroughly for 30 min, after which 5 mL of the different concentrations of CW emulsion was added dropwise to the PVA/GLY solution with increasing temperature to 120 °C and continuous stirring in a water bath. The mixture was stirred for 5 min to ensure its homogeneity. The stirring was stopped and allowed to cool prior to casting onto Petri dishes. The unplasticized control sample PVA/CW, and the plasticized samples PVA-CW-G1:3, PVA-CW-G2:6, PVA-CW-G4:9, and PVA-CW-G6:12, were kept for 2 days in a ventilated enclosure and cured for 24 h at room temperature in a desiccator. After curing, the films were peeled off and analyzed in triplicate.

### 2.3. Tensile Properties

The mechanical properties of the polymer/wax films were investigated through a digital tensile tester [14]. These properties are essential for determining the films strength, flexibility, and stiffness. The measure of maximum stress that a material can withstand when stretched is the tensile strength. Elongation indicates the flexibility of a film [8]. Young’s modulus represents the stiffness or rigid modulus of the material [13]. Instron model 5542 universal testing machine was used to evaluate these properties following the ASTM D-638 standard protocol. The average thickness of the films was obtained using a Micrometer [10].

### 2.4. Functional Groups

Fourier transform infrared analysis of the films was performed using a Thermo Scientific Nicolet 8700 within the range of 4000–400 cm^−1^ during 64 scans at 4 cm^−1^ resolution [10]. Data acquisition and analysis were performed using the OMNIC Specta^®^ software (v9.50.1.4).

### 2.5. Surface Morphology

SEM analysis from a previous study was applied [13]. The samples were then coated with gold using a sputter coater (Quorum Technologies Ltd., Laughton, UK). The surfaces of the films were observed using scanning electron microscopy (SEM, JEOL JSM-7600F, Tokyo, Japan) at an accelerating voltage of 10 kV.

### 2.6. Film Solubility

The method described in previous work was followed in this study [15]. Small pieces of the film (2 cm × 2 cm) were placed in an oven at 105 °C for a day to obtain the initial dry mass (*M_i_*) of the film. Thereafter, the films were placed in a beaker containing distilled water at room temperature. The final weights (*M_f_*) were determined after drying. The solubility of the films was calculated as follows:

(1) $$Solubility\left(\%\right)=\frac{M_{i}-M_{f}}{M_{i}}\times 100\%$$

### 2.7. Contact Angle

DSA 100, KRUSS, Hamburg, Germany was used to capture images of the water drops on the surface of the film samples. This was analyzed using ADVANCE^®^ software (v3.18.5). For each film sample, the contact angle was measured three times, and the mean value was used for analysis. The parameter was based on a sessile drop of 2°, temperature of the environment set at 23 °C, needle width of 0.525 mm, and drop size of 5 mL [13].

### 2.8. Thermal Gravimetric Analysis (TGA)

The stability of the samples was investigated using a TGA 550 instrument (TA Instruments, New Castle, DE, USA). The aluminum pans were first tarred for 10 min before loading the samples in the pans. Film was heated from 20 °C to 600 °C at a flow rate and heating rate of 60 mL/min and 20 °C/min, respectively, under nitrogen gas. Film sample weight percentage was plotted as a function of temperature in the TRIOS software (v5.1.1.46572).

### 2.9. Differential Scanning Calorimetry

The DSC was used to measure glass transition temperature (T_g_), melting temperature (T_m_), and degradation temperature (T_d_) in accordance with previous study [16]. Film samples were heated between 20 °C to 150 °C at a 10 °C/min heating rate in a sealed aluminum pan under nitrogen gas at a flow rate of 50 mL/min were determined from the DSC thermograms.

### 2.10. Data Analysis

The statistical analysis was conducted using Excel software (v2409). Data was collected in triplicates. The difference in means was tested using one-way analysis of variance (ANOVA), followed by Tukey’s multiple comparison test with statistically significant at *p* ≤ 0.05.

## 3. Results and Discussion

### 3.1. Functional Group Study

Fourier transform is used to study changes in a film’s structure when in contact with infrared in the electromagnetic spectrum. This involves the identification and characterization of the chemical bonds and functional groups that are present [17]. It also provides information on physical interactions and compatibility when more than one material is involved. Poly(vinyl alcohol) and PVA containing varying percentages of glycerol and wax were analyzed.

**Figure 1:**
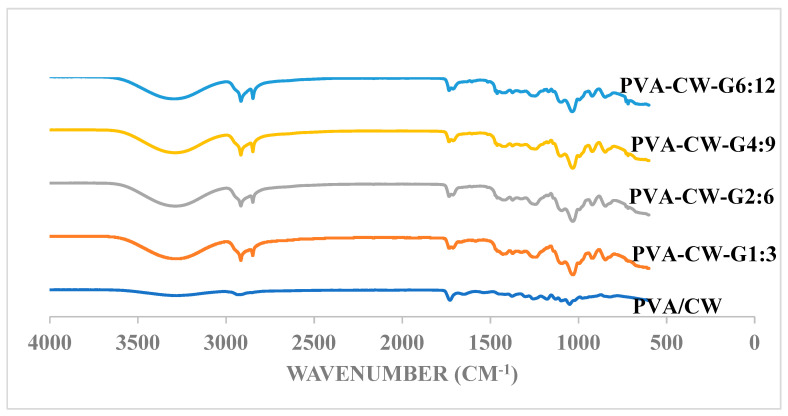
FTIR Spectra for all samples.

FTIR spectra analysis in Figure 1 shows broadbands between 3000 and 3500 cm^−1^. These bands indicate the O-H functional group. The increased plasticizer concentration resulted in a broader peak and a shift in wavenumber. This shift implies the weakening of the hydrogen bond of the film and suggests good interaction among the functional groups of PVA, CW, and glycerol. The observation from this study is concurrent with previous reports where glycerol and wax incorporated in a high amylose starch blend showed a similar trend [18]. The vibrational band observed at 2900 cm^−1^ indicates the stretching of C-H from the alkyl groups. Owing to the addition of glycerol, two peaks were observed at 2915 and 2848 cm^−1^, respectively, compared to PVA/CW film, with a single peak at 2932 cm^−1^. The peak intensity decreased as the glycerol percentage increased, indicating a decrease in C–H within the film. The peaks at 1700 cm^−1^ were due to the presence of a C=O bond in the wax. The peaks between 1000 and 110 cm^−1^ were due to the stretching of the C-O group, which was present in all samples. These peaks became sharper and shifted to lower wavelengths as the glycerol ratio of the sample increased. This can be attributed to an increase in the O-H from glycerol. The broad peak progressively shifted to a higher wavelength region and became sharper and more defined as the glycerol weight percentage increased. The findings from the FTIR analysis of the sample showed the possible physical interaction and compatibility between glycerol and the PVA/wax blend.

### 3.2. Tensile Strength of PVA Films

**Figure 2:**
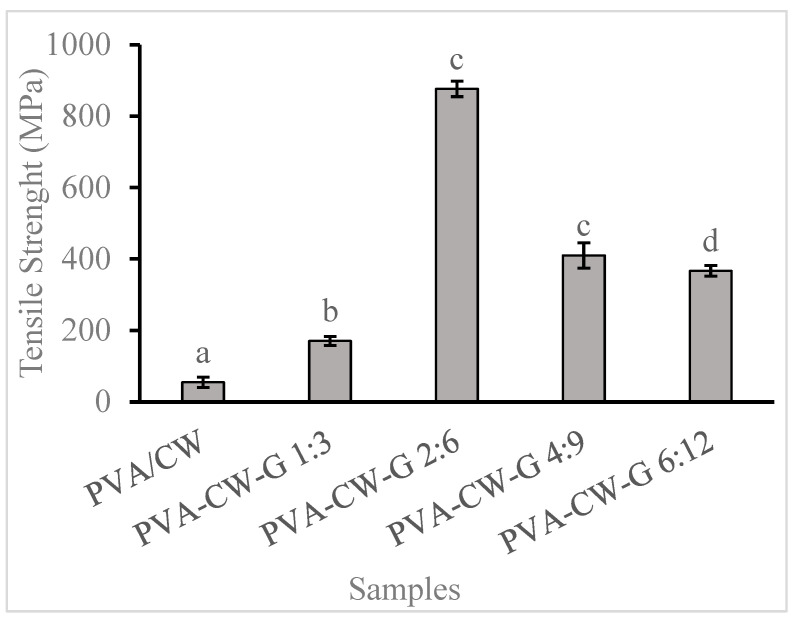
Film tensile strength. Note: Different letters (a, b, c, and d) indicate that the means are significantly (*p* < 0.05) different for film tensile strength.

The tensile strength of composite material is the ability to withstand pulling forces. The result in Figure 2 shows that 2.5% glycerol significantly increased the film’s tensile strength by 20% compared to the unplasticized film. An explanation for this is the domination of weak interactions of PVA with polar groups in CW over hydrogen bonds between PVA–plasticizer attraction. This indicates that the PVA/CW films became more resistant to stress when glycerol was added. A similar result when glycerol was added to PVA films has been reported [19]. Also, a significant decrease in tensile strength with increased glycerol concentration of the PVA-CW-G 6:12 blend compared to the PVA-CW-G 2:6 blend could be attributed to an increase in the amount of CW leading to excessive substitution of PVA/glycerol chains with weaker PVA-wax interactions. In another study, an increase in glycerol with oxidized banana starch/PVA blend resulted in a decline in the film’s strength [20]. A similar trend has been observed by previous researchers, where increased glycerol concentration affected the tensile strength [21,22,23]. The stress-strain curve in Figure S1 shows the relationship between films tensile strength and flexibility.

### 3.3. Film Elongation

**Figure 3:**
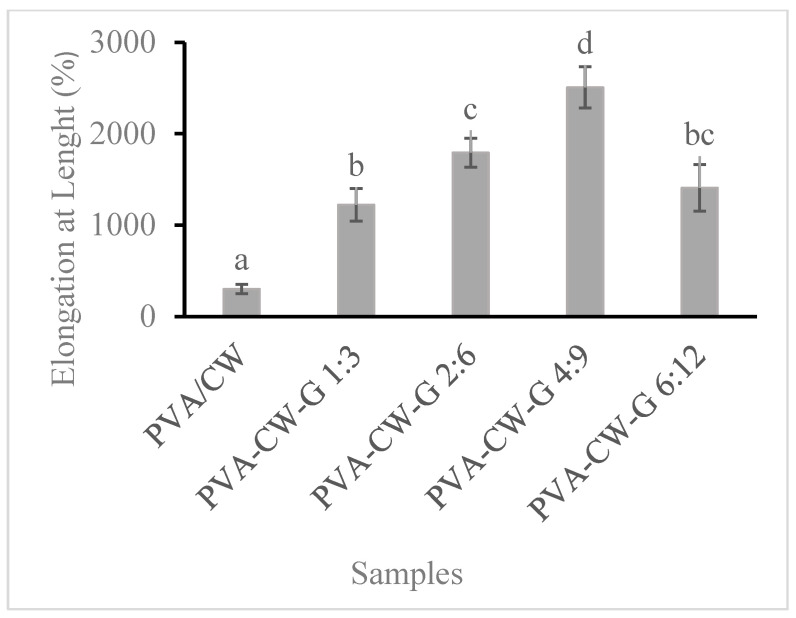
Film’s elongation. Note: Different letters (a, b, c, and d) indicate that the means are significantly (*p* < 0.05) different for films elongation.

Elongation at break is a desirable mechanical property in packaging materials because it indicates improved flexibility and durability of the material. It allows the packaging to withstand stretching and bending forces without tearing or breaking. To investigate the effect of the plasticizer on the film’s elongation, varying glycerol concentration was added to the PVA/CW mix. Figure 3 shows that the addition of glycerol (1.5–3.5%) significantly increased (*p* < 0.05) the film’s elongation, indicating an interaction between glycerol and PVA/CW. A similar increase in biopolymer elongation was reported with the addition of plasticizer [24,25]. The previous explanation stated that small molecular size glycerol occupies the space between PVA/CW, resulting in increased chain mobility and an enhanced plasticizing effect. However, a reduction in the film’s flexibility with 4.5% glycerol was observed. This reduced elongation observed in the film could be due to phase separation, which results from the increased concentration of CW. In a study of the thermal and tensile properties of sugar palm, there was a report of decreased elongation of SPS films at a higher concentration of plasticizer (45% *w*/*w*) [25]. The decrease in film elongation in this study was attributed to phase separation within the polymer matrix.

### 3.4. Film Young’s Modulus

**Figure 4:**
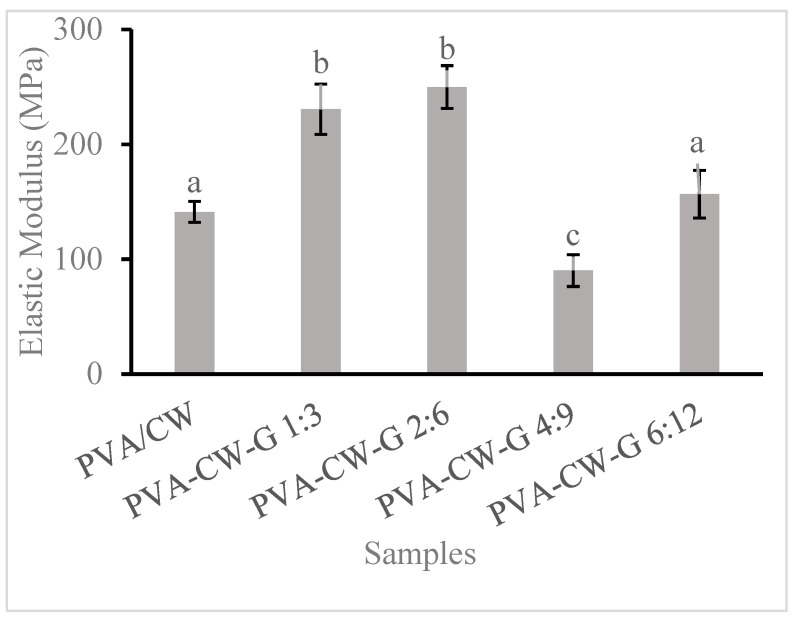
Film Young’s modulus. Note: Different letters (a, b, and c) indicate that the means are significantly (*p* < 0.05) different for films modulus.

Young’s modulus determines the film’s toughness and shows the relationship between tensile stress and strain. Figure 4 shows that the ratio of glycerol or wax in film composite affected its stiffness. Films with wax/glycerol ratios of 1:3 and 2:6 showed increased modulus. However, an increase in glycerol to the composite disrupted and weakened the intermolecular forces in the PVA matrix because of the inclusion of hydrophilic functional groups in glycerol, which caused Young’s modulus of the PVA-CW-G 4:9 and PVA-CW-G 6:12 films to drop. A study of corn starch films with varied glycerol concentrations revealed a similar result to this finding [26]. The addition of glycerol promoted a more compact polymer network without pores or cracks, unlike film formed without glycerol. The report from this study is similar to the findings from a previous study [27]. The report from the study shows that the addition of glycerol to a cassava–starch–beeswax mix makes the films more compact without cracks and pores. This present study revealed that glycerol is a compatible plasticizer to PVA/wax and interacts fully with the polymer by distributing itself consistently within the polymer matrix. This was confirmed by FTIR results.

### 3.5. Scanning Electron Microscope

SEM is used to investigate the surface morphology and characteristics of a material. A JEOL JSM-7600F field-emission SEM analysis of film samples was conducted to examine the film microstructure.

**Figure 5:**
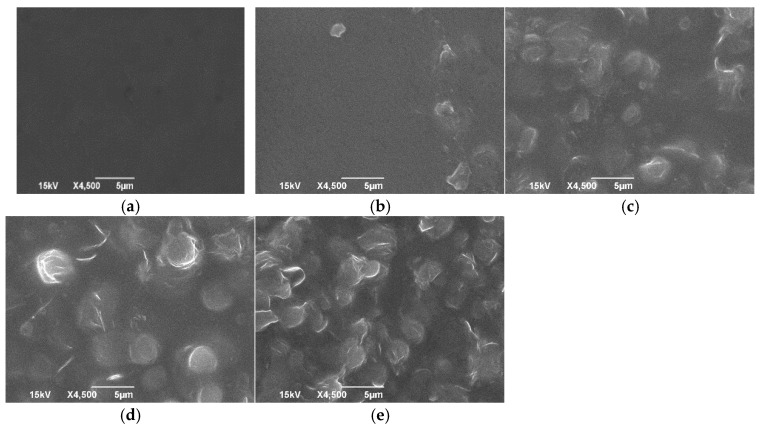
SEM micrographs of the films (**a**) PVA-CW; (**b**) PVA-CW-G 1:3; (**c**) PVA-CW-G 2:6; (**d**) PVA-CW-G 4:9; (**e**) PVA-CW-G 6:12.

SEM images are shown in Figure 5. Significant changes were observed in the microstructures of films with the addition of glycerol. This revealed that as plasticizer concentration increased from 1.5% to 4.5%, the roughness of the film surface increased. Increasing the CW concentration made the films surface rougher and uneven. A report from a previous study attributed this uneven surface structure to different drying rates between the layers of the emulsion during the film’s formation. Also, CW had a higher melting point temperature (60–80 °C) than the film’s drying temperature (20–24 °C). This may cause rapid crystallization when emulsion temperature decreases [28].

### 3.6. Water Resistance Analysis of Films

#### 3.6.1. Water Contact Angle Test

The water contact angle is a measure of how effectively a surface repels water. Higher angles (>90°) indicate more hydrophobic surfaces, while angles (<90°) reveal hydrophilic surfaces. Understanding the surface wettability of these films could provide valuable insights into how the addition of glycerol affects the film’s tensile properties. Similarly, their surface characteristics, which are crucial for various applications such as packaging, coatings, and biomedical materials were also improved [29].

**Figure 6:**
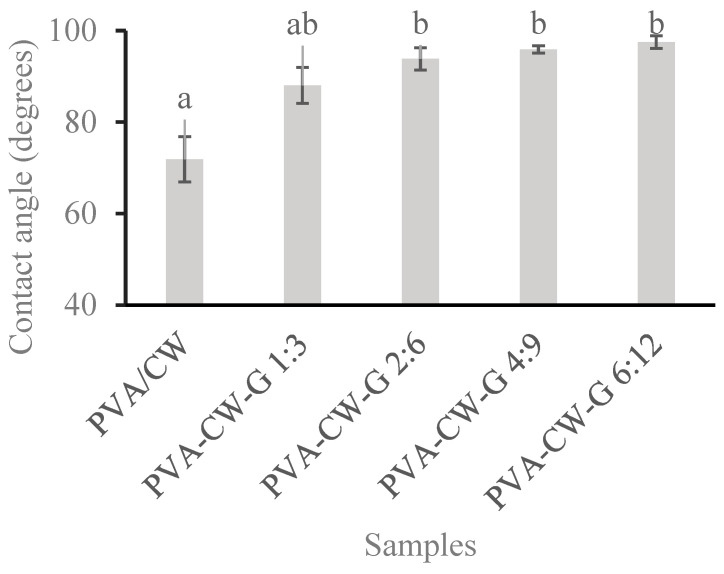
Film water contact angle. Note: Different letters (a, b) indicate that the means are significantly (*p* < 0.05) different for films contact angle.

Figure 6 shows the contact angles of PVA samples 2:6, 4:9, and 6:12 were higher than those of unplasticized films. This can be attributed to strong hydrogen bond interaction between PVA and glycerol, leading to the exposure of more COOH groups of CW on the film’s surface. As a result, this hydrophobic group migrated to the film’s surface, and an increased contact angle was observed. This is also in line with FT-IR results [8,18,29]. The control film and PVA-CW-G 1:3 had angles (>90°), indicating the hydrophilic films. From Figure 6, we can deduce that the hydrophobicity of the resulting film increased with wax and glycerol increase compared to that of the control.

#### 3.6.2. Solubility

Assessing a film’s solubility is crucial for understanding its environmental impact, evaluating its biodegradability, improving its practical uses, and refining film manufacturing and handling methods. Additionally, it provides important insights into the interaction between films and water, and aiding the development of eco-friendly and tailored films.

**Figure 7:**
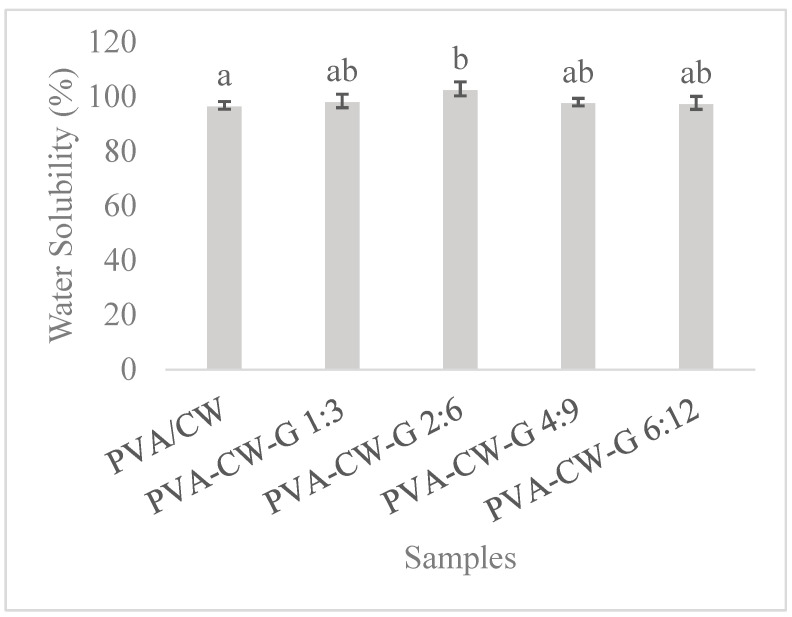
Film solubility. Note: Different letters (a, b) indicate that the means are significantly (*p* < 0.05) different for films solubility.

PVA films are water-soluble [30]. Hydrophobic materials such as wax are often incorporated into these films to reduce the overall solubility of the resulting material. The solubilities of composite films are shown in Figure 7. This result clearly confirms the dissolution of plasticized films in water compared to unplasticized PVA/CW samples. This could result from increased glycerol concentration in PVA, which resulted in hydrophilicity of films. Consequently, increasing glycerol concentration in the blends led to an increase in the hydroxyl functional groups, which enhanced the solubility of the blend films. This observation aligns with previous findings [20,31].

### 3.7. Thermal Analysis

Polymeric materials contain organic macromolecules and low molecular weight organic compounds within the polymer matrix. Plasticizers are sometimes added to polymeric materials to modify the polymer matrix’s three-dimensional structure. This modification leads to a reduction in intermolecular attractive forces and increased polymeric chain’s free volume and mobility. TGA is a crucial technique for examining the degradation and thermal characteristics of polymer-based materials, which are utilized in industrial and scientific contexts for bio-composite development [18,32].

**Figure 8:**
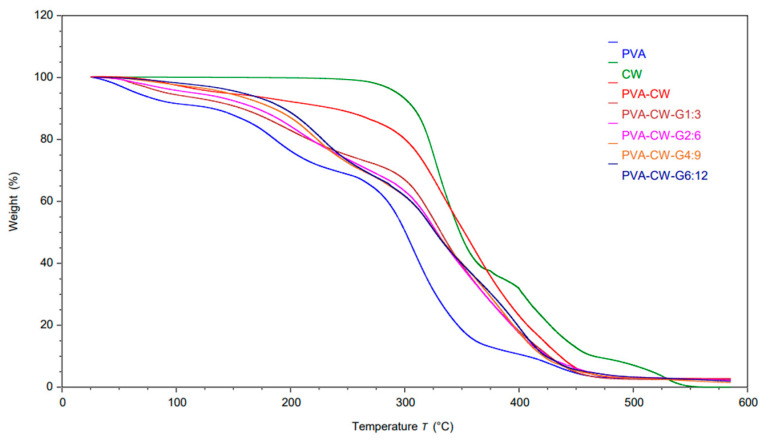
Thermogravimetric graph of PVA/CW plasticized films.

To assess thermal stability and corresponding degradation stages in relation to temperature, TGA was performed on PVA, CW, PVA/CW and PVA/CW/Glycerol blends. Figure 8 displays the thermogravimetric (TGA) for unplasticized and plasticized samples. This reveals the PVA film’s degradation in stages. The findings from this study align with the reports observed in most PVA-based films from previous studies [18,33,34,35,36]. The commencement of thermal breakdown in films occurred at temperatures below 100 °C. This initial weight reduction can be attributed to the evaporation of loosely attached water molecules in the films. The report from the previous study is consistent with this finding [37]. Higher glycerol concentrations notably reduced the weight loss rate of plasticized films compared to unplasticized ones at a constant temperature. This observation contrasts with previous research with reports of a significant increase in thermal degradation with rising glycerol concentration [25,38]. This was attributed to glycerol–polymer molecular interactions weakening the strong intermolecular bonds between polymer molecules, thus lowering the thermal resistance of glycerol-plasticized films. However, the result from this study indicates otherwise, possibly due to the presence of wax in the polymer matrix. The wax might mitigate the effect of glycerol’s interaction with PVA, strengthening intermolecular bonds and enhancing the thermal resistance of plasticized films. Loss of glycerol molecules occurred between 260 °C and 450 °C [39]. These results show an increasing plasticizer concentration elevates the degradation of plasticized films unlike the control film (55.11%) at a constant temperature. The final stage of film degradation, characterized by mass loss, occurred at temperatures exceeding 450 °C. This weight reduction was attributed to the breakdown and disintegration of carbon chains within the polymer matrix. Notably, the film containing the highest glycerol content exhibited the greatest mass residue (3.3%) at 500 °C. Conversely, the film with the lowest plasticizer concentration displayed the smallest mass residue (2.56%), which was even less than that of the control sample (2.82%). This shows that the addition of glycerol and CW to PVA slows down thermal degradation of the films.

### 3.8. Differential Scanning Calorimetric Analysis

Analyzing T_g_, T_m_ and T_d_ through DSC is crucial as these factors influence the thermal properties, as well as the physical and stability of biopolymer. T_g_ marks the onset of molecular mobility, representing the point of biopolymer transition when heated. This investigation examined the T_g_ and T_m_ values of plasticized and unplasticized PVA/CW films to evaluate how varying glycerol concentrations affect these parameters. The DSC thermograms consistently displayed two transitions: one related to glass transition and another associated with melting. The presence of a single glass transition peak indicates compatibility between the plasticizer and PVA/CW films [40]. Table 2 presents the DSC parameters of PVA/CW films.

In the present study, the decrease in T_g_ values of PVA/CW films is insignificant as plasticizer concentrations increased. The increase in glycerol concentration leads to an increase in the free volume and mobility of molecules, changing the physical structure of the PVA/CW film, which agrees with the free volume theory of plasticization. This is also evident with the decrease in the T_g_ values from 55.39 °C to 53.01 °C. The obtained results agree with numerous findings that reported the decrease of T_g_ as plasticizers are incorporated into polymer-based films [35,41].

## 4. Conclusions

PVA/CW films are brittle with many visible cracks and are not easily peeled from the casting surface. Hence, the addition of glycerol helped to overcome brittleness and enhance the film’s flexibility. Improvements in the flexibility and strength of the blend, coupled with the reduced wettability of the films, led to this conclusion. Although the films showed vulnerability to water penetration at higher concentrations of glycerol, they exhibited enhanced mechanical properties and an elevated water contact angle. Overall, PVA-CW-G4:9 shows the highest performance after the analysis of the results. This study also revealed that the addition of glycerol to the polymer blend is distributed within the polymer matrix, thereby optimizing the overall performance of the films. The mass residue at a temperature above 500 °C increased with glycerol addition. These results imply that glycerol could be a useful additive for applications in food packaging and wrapping, offering enhanced functionality and durability to these materials. Further studies on the enhancement of the functional characteristics of PVA films with different plasticizers and bioactive agents may pave the way for the development of more sustainable and efficient packaging solutions.

## Figures and Tables

**Table 1:** Blend composition in terms of poly(vinyl alcohol) (PVA), carnauba wax (CW), glycerol (G), stearic acid (SA), and polysorbate-20.

Blends	PVA	Glycerol	Polysorbate-20	CW/SA
PVA/CW	2.5	-	0.3	0.1
PVA-CW-G1:3	2.5	1.5	0.3	0.5
PVA-CW-G2:6	2.5	2.5	0.3	1.0
PVA-CW-G4:9	2.5	3.5	0.3	2.0
PVA-CW-G6:12	2.5	4.5	0.3	5.0

**Table 2:** Thermal properties by differential scanning calorimetry.

Blends	T_g_ (°C)	T_m_ (°C)	T_d_ (°C)
PVA/CW	55.39 ± 1.7 ^a^	110.04 ± 3.2 ^d^	146.24 ± 1.5 ^f^
PVA-CW-G1:3	51.45 ± 0.15 ^b^	77.65 ± 0.5 ^e^	80.21 ± 2.7 ^g^
PVA-CW-G2:6	51.95 ± 0.18 ^b^	77.99 ^e^ ± 0.8	80.96 ± 3.5 ^g^
PVA-CW-G4:9	52.58 ± 1.4 ^c^	77.39 ± 0.6 ^e^	81.39 ± 1.9 ^g^
PVA-CW-G6:12	53.01 ± 0.56 ^bc^	77.60 ± 0.4 ^e^	81.45 ± 2.2 ^g^

Note: Different letters indicate that the means are significantly (*p* < 0.05) different for films thermal properties.

## Data Availability

Data are contained within the article.

## Supplementary Materials

The following supporting information can be downloaded at: https://www.mdpi.com/article/10.3390/polym16213024/s1, Figure S1: Stress-strain curve graph of unplasticized and plasticized films.
