# Supplementary figures and images for: Mechanical and Thermal Characteristics of Films from Glycerol Mixed Emulsified Carnauba Wax/Polyvinyl Alcohol
Source: Polymers (Basel). 2024 Oct 28;16(21):3024. doi: 10.3390/polym16213024 (PMC11548645; doi:10.3390/polym16213024)

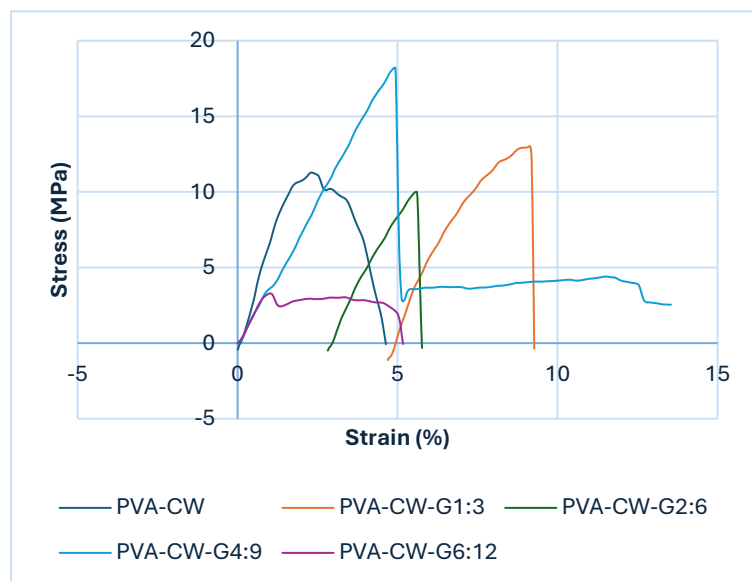

Figure S1: Stress-strain curve graph of unplasticized and plasticized films.

Supplement: Supplementary file 1 [file polymers-16-03024-s001.zip › polymers-3249129-supplementary.pdf]
